# Oligo-carrageenan kappa increases glucose, trehalose and TOR-P and subsequently stimulates the expression of genes involved in photosynthesis, and basal and secondary metabolisms in *Eucalyptus globulus*

**DOI:** 10.1186/s12870-019-1858-z

**Published:** 2019-06-17

**Authors:** Silvia Saucedo, Alberto González, Melissa Gómez, Rodrigo A. Contreras, Daniel Laporte, Claudio A. Sáez, Gustavo Zúñiga, Alejandra Moenne

**Affiliations:** 1 Faculty of Chemistry and Biology, University of Santiago of Chile, Santiago, Chile; 2 Facultad de Ciencias Agrarias, Universidad Técnica Estatal de Quevedo, 63 Quevedo, Ecuador; 3 Laboratory of Coastal Environmental Research, Center of Advanced Studies, University of Playa Ancha, Viña del Mar, Chile

**Keywords:** *Eucalyptus*, Gene expression, Glucose, Growth, OC kappa, Photosynthesis, TOR kinase, Trehalose

## Abstract

**Background:**

It has been previously shown that oligo-carrageenan (OC) kappa increases growth, photosynthesis and activities of enzymes involved in basal and secondary metabolisms in *Eucalyptus globulus.* However, it is not known whether OC kappa may induce the activation of TOR pathway and the increase in expression of genes encoding proteins involved in photosynthesis and enzymes of basal and secondary metabolisms.

**Results:**

*E. globulus* trees were sprayed on leaves with water (control) or with OC kappa 1 mg mL^− 1^, once a week, four times in total, and cultivated for 17 additional weeks (21 weeks in total). Treated trees showed a higher level of net photosynthesis than controls, beginning at week 3, a higher height, beginning at week 9, and those differences remained until week 21. In addition, treated trees showed an increase in the level of glucose beginning at week 1, trehalose at weeks 1–3, and in TOR-P level at week 1–2. On the other hand, transcripts encoding proteins involved in photosynthesis, and enzymes involved in glucose accumulation, C, N and S assimilation, and synthesis of secondary metabolites began at weeks 3–4 and with additional peaks at weeks 5–6, 8–11,13–14 and 17–19. Thus, OC kappa induced initial increases in glucose, trehalose and TOR-P levels that were followed by oscillatory increases in the level of transcripts coding for proteins involved in photosynthesis, and in basal and secondary metabolisms suggesting that initial increases in glucose, trehalose and TOR-P may trigger activation of gene expression.

**Conclusions:**

The stimulation of growth induced by OC kappa in *E. globulus* trees is due, at least in part, to activation of TOR pathway and the increase in expression of genes encoding proteins involved in photosynthesis and enzymes of basal metabolism.

**Electronic supplementary material:**

The online version of this article (10.1186/s12870-019-1858-z) contains supplementary material, which is available to authorized users.

## Background

It is now well known that growth and development in mammals, nematodes yeast, plants and algae is controlled by the kinase Target of Rapamycin (TOR) [1–3]. TOR is a phosphoinositol-related kinase (PIK) having serine/treonine protein kinase activity and is a key regulatory kinase of the TOR pathway [1, 2, 4]. TOR kinase is a large protein constituted by several domains: a N-terminal Huntingtin, Elongation Factor 3, Regulatory Subunit A of PPA2, TOR1 (HEAT) domain containing several HEAT repeats which are constituted by 37–47 amino acids forming two *α*-helices and a solenoid structure that is involved in protein-protein interactions [5]. Contiguous to HEAT domain, is FRAP, ATM; TTRAP (FAT) domain that is present in most PIK and is involved in protein-protein interactions [6]. After FAT, is located FRB FKBP-Rapamycin-Binding (FRB) domain that binds to FKBP12-rapamycin complex that inhibit TOR activity [7, 8]. Contiguously is located the catalytic domain (CD) that interacts with Lethal with SEC13 protein 8 (LST8) regulatory protein [9] and with RAPTOR [10, 11]. After CD, is located the C-terminal FAT domain (FATC) that is redox-sensitive and binds to membranes [12, 13]. In mammals, TOR kinase is inhibited by nanomolar concentrations of the macrolide rapamycin produced by the bacteria *Streptomyces hygroscopicus* [14]. In contrast, TOR kinases are only moderately sensitive to rapamycin in plants [15, 16]. In this respect, it has been shown that when FKBP12, a prolyl isomerase, is overexpressed or replaced by human or yeast FKBP12 in *Arabidopsis thaliana*, TOR kinase becomes sensitive to rapamycin [17, 18].

In mammals, TOR is encoded by a single gene corresponding to a large protein of around 280 kDa that is activated by phosphorylation in Treo 2446, Ser 2448, Ser 2481 and Ser1261 [19, 20]. TOR pathway is activated by growth factors, pro-inflammatory cytokines, insulin, glucose, amino acids such as glutamine and leucine, and lipids; the latter leads to an increase in anabolic reactions, cell division and growth [2, 21]. In mammals, TOR kinase can interact with proteins RAPTOR, LST8 and FKBP12 to form complex TORC1, which is sensitive to rapamycin, and it can also interact with RICTOR, LST8 and SIN1, to form TORC2, which is insensitive to rapamycin [22–24]. TORC1 regulates the equilibrium among anabolism and catabolism, cell proliferation and temporal growth whereas TORC2 modulates cytoskeleton structure, spatial cell growth, cell polarity and apoptosis [24]. It is now clearly established that TORC1 is activated by small GTPases such as Rheb and Rag which binds to the catalytic site of TOR and to LST8 [2] and that these GTPases can be activated by hormones, growth factors, glucose and some amino acids [2].

In plants*,* TOR is encoded by a single gene that is essential for post-embryonic development [25] and corresponds to a protein of around 250 kDa, 39% identical in its amino acid sequence to human TOR [2, 3]. Antibodies anti-human TOR-P Ser2448 recognize phosphorylated TOR (TOR-P) in Ser2424 in *Arabidopsis* TOR [3, 26, 27]. In plants, TOR is activated by sucrose, glucose and fructose feeding [28–31]. In addition, glucose is rapidly converted into glucose − 6-P (G6P) and glucose-1-P (G1P) in plants [31]. In addition, trehalose 6-P is a precursor of trehalose and the increase in T6P lead to an increase in trehalose enhancing tolerance to abiotic stress in rice [32]. Moreover, it has been shown that G1P, G6P and T6P, as well as ribose 6P, directly inhibits SnRK1 [33–36]), a kinase that phosphorylates RAPTOR inhibiting TOR activity [11]. Thus, the increase in glucose may lead to the increase in G1P, G6P and T6P that may inhibit SnRK1 activating TOR kinase and TOR pathway. Recently, it has been shown that auxin activate the cell surface ABP1-TMK auxin-sensing complex which in turn activates ROP2 GTPase [37] and that the activation of ROP2 leads to activation of TOR which, in turn activate s6K and translation [27]. Therefore, TOR and SnRK1 are kinases that can sense the nutritional status of the cells and snRK1 has an antagonistic action regarding TOR kinase.

It has been shown that activation of TOR pathway in *A. thaliana* results in an increased expression of genes encoding enzymes involved in anabolic reactions, such as those related to the syntheses of proteins, amino acids, RNA, DNA and cell wall, as well as synthesis of enzymes involved in glycolysis, TCA cycle and proteins of mitochondrial electron transport chain [16, 30, 38]. Moreover, it has been shown that inhibition of TOR pathway using the inhibitor of TOR kinase AZD8055 leads to a decrease in transcripts encoding proteins involved in photosynthesis, chlorophyll synthesis and C assimilation [39, 40] indicating that the activation of TOR pathway leads to the increase photosynthesis and basal metabolism. Furthermore, the activation of TOR pathway down-regulates expression of genes coding for catabolic enzymes involved in protein, amino acids and lipid syntheses, and those related to starch degradation, autophagy and glyoxylate cycle [30]. In addition, activation of TOR pathway also increased the expression of genes coding for enzymes involved in secondary metabolism and defense responses, such as those that synthesize glucosinolates [30]. Similarly, TOR pathway mediates the increase in the level of phenylpropanoid compounds (PPCs) and glucosinolates in *A. thaliana* [38]. In this sense, it has been shown in yeast that TOR-P can enter into the nucleus and bind RNA polymerase II leading to activation of gene expression [41].

Marine algae oligo-carrageenans (OCs) enhance growth and defense responses in terrestrial plants [42–44]. OCs kappa, lambda and iota are obtained by acid hydrolysis of pure carrageenans kappa, lambda and iota, respectively, and displayed a DP = 20–25 [42]. It was initially determined that OCs kappa, lambda and iota applied on plant leaves at a concentration of 1 mg mL^− 1^, once a week, four times in total, mediated an increase in height and plant biomass in tobacco plants (var. Xhanti) cultivated in control conditions as well as tobacco plants (var. Burley) cultivated outdoors for four months [45]. In addition, OCs kappa, lambda and iota applied at a concentration of 1 mg mL^− 1^, once a week, four times in total, induced and increase in height, trunk diameter, net photosynthesis, and in the levels of PPCs and essential oils in *Eucalyptus globulus* cultivated in the field for three years [46]. On the other hand, it was shown that OC kappa mediated an increase in the synthesis of reducing compounds such as NADPH, ascorbate (ASC), and glutathione (GSH), as well as increased activities of thioredoxin reductase and thioredoxin in *E. globulus* trees cultivated for four months outdoors. In addition, OC kappa increases the activity of enzymes involved basal metabolism, C, N and S assimilation, purine and pyrimidine syntheses, and in the activities of Krebs cycle enzymes in *E. globulus* trees [47]. Furthermore, OC kappa increased the level of growth-promoting hormones such as auxin, gibberellin and cytokinines in *E. globulus* trees [48] as well as in pine trees [49]. In addition, OC kappa increases the amount of volatile terpenes, and new terpenes having potential anti-pathogenic activities in *E. globulus* trees [50]. Thus, OC kappa mediated an increase in net photosynthesis, basal and secondary metabolisms in *E. globulus* trees.

In order to analyze whether the increase in photosynthesis, and in basal and secondary metabolisms observed in *E. globulus* trees treated with OC can be due to increased expression of genes encoding proteins involved in photosynthesis and enzymes of basal and secondary metabolisms, the level of transcripts encoding subunits of enzymes that increase glucose level, proteins of PSII and PSI, an enzyme involved in chlorophyll synthesis, and enzymes of basal and secondary metabolism were analyzed for 21 weeks. In addition, the levels of glucose, trehalose and TOR-P were analyzed in order to determine whether these increases precede the increase in gene expression and, thus, may be involved in the stimulation of gene expression.

## Results

### OC kappa-induced increases in levels of glucose and trehalose which precede the increase in photosynthesis and growth

**Fig. 1:**
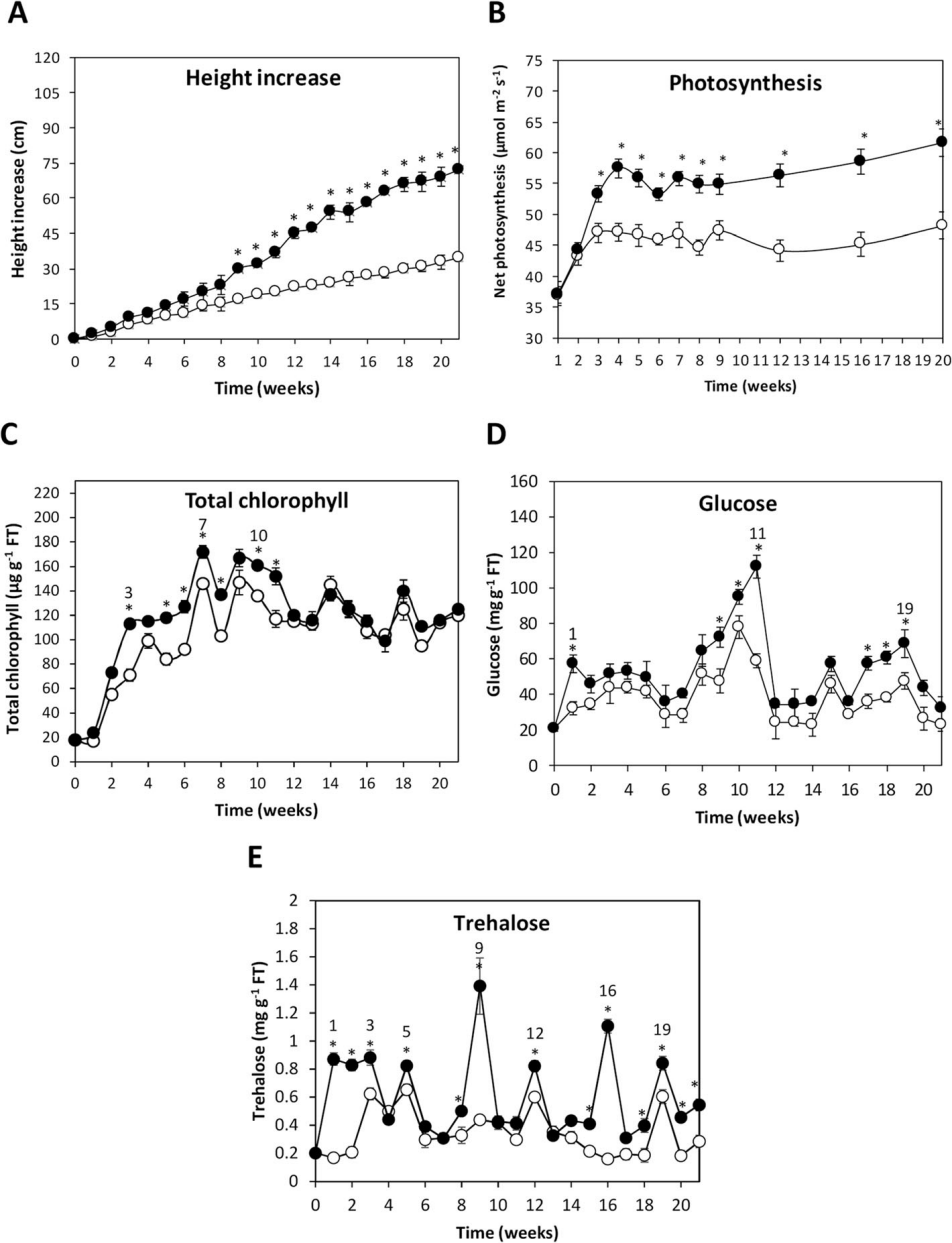
Height, photosynthesis and sugar levels in *E. globulus* treated with OC kappa. Increase in eight (**a**), net photosynthesis (**b**), level of total chlorophyll (**c**), glucose (**d**) and trehalose (**e**) in control (open circles) and in *E. globulus* trees treated with OC kappa at 1 mg mL^− 1^ (black circles). Height is expressed in centimeters, net photosynthesis in micromoles per meter per second, level of total chlorophyll in micrograms per gram of fresh tissue and the level of glucose and trehalose are expressed in milligram per gram of fresh tissue. Numbers over circles highlight weeks when the most important peaks were observed. Within each experimental week, asterisks (*) indicate when there are significant differences (*p* < 0.05) between OC kappa-treated and control trees. Circles represent the mean value of three independent triplicates ± SD

*E. globulus* trees treated with OC kappa showed significant higher increase in height compared to controls, which started at week 9 and became more evident with time until week 21 (Fig. 1a). Treated trees showed an average height of 72 cm at week 21, whereas control trees showed an average height of 35 cm, indicating 105% higher increase in height in OC kappa-treated trees with respect to controls. In addition, trees treated with OC kappa showed an increase in net photosynthesis beginning at week 3, that remained until week 21, and was higher in 33% than controls (Fig. 1b). In addition, a higher level of total chlorophylls starting at week 3 and remaining until week 11 were observed; the following week, the levels of total chlorophylls decreased to reach control levels in a continuing pattern until the end of the experiments (Fig. 1c). Furthermore, treated trees showed in general higher levels of glucose compared with controls; these differences were significant at weeks 1, from weeks 9–11, and from weeks 17–19 (Fig. 1d). Treated trees showed a trend of higher levels of trehalose if compared with controls with maximal level at weeks 1–3, 5, 8–9, 12, 15–16 and 18–21 (Fig. 1e).

### OC kappa-induced levels of transcripts encoding enzymes involved in glucose accumulation

**Fig. 2:**
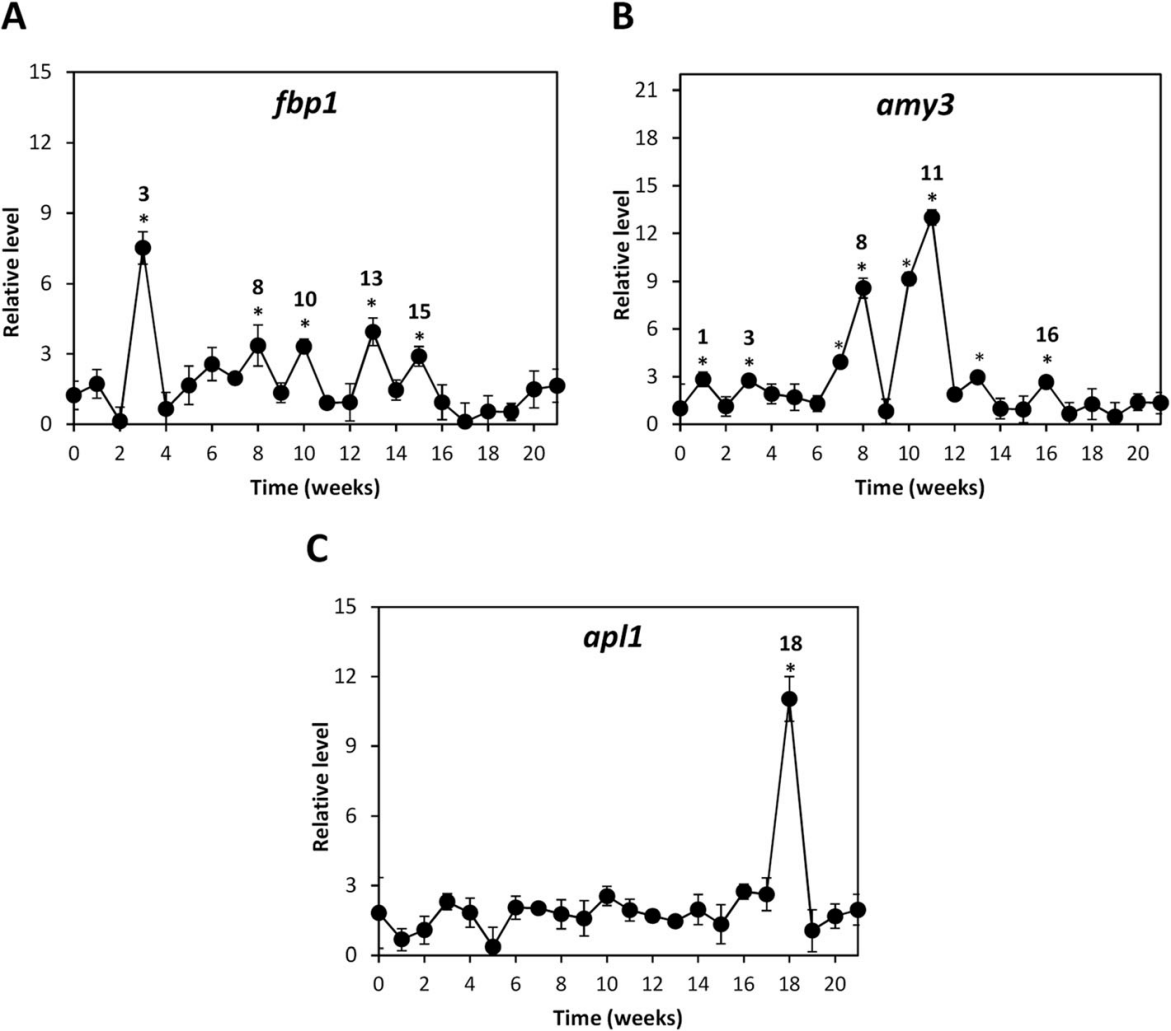
Level of transcripts encoding enzymes involved in glucose metabolism in *E. globulus* treated with OC kappa. Relative level of transcripts encoding enzymes fructose-1,6-bisphosphatase (*fbp1*, **a**), amylase (*amy3*, **b**), and ADP-glucose pyrophosphorylase (*apl1,* **c**) in control and in *E. globulus* trees treated with OC kappa at 1 mg mL^− 1^. Asterisks (*) represent significant differences (*p* < 0.05) between the level of transcripts at a certain week compared with the expression at the beginning of the experiments (week 0). Relative level of transcripts is expressed as 2^-ΔΔCt^. Circles represent the mean value of three independent triplicates ± SD

In order to analyze the reasons explaining the increases in the level of glucose induced by OC kappa, the level of transcripts encoding the enzymes fructose-1,6-bisphosphatase involved in glucose synthesis, *fbp1*; α-amylase 3 involved in starch degradation and production of glucose, *amy3*; and ADP-glucose pyrophosphorylase involved in starch synthesis and glucose accumulation, *apl1*, were detected. Trees treated with OC kappa showed an increase in the level of *fbp1* transcripts at weeks 3, 6, 8, 10, 13 and 15 with respect to controls (Fig. 2a). Transcript levels of *amy3* peaked at weeks 1, 3, 7–8, 10–12 and 16, compared to controls; peaks were the highest at weeks 7 and 11 (Fig. 2b). *Apl1* transcripts increased only at week 18 (Fig. 2c).

### OC kappa-induced increase in the level of TOR-P

**Fig. 3:**
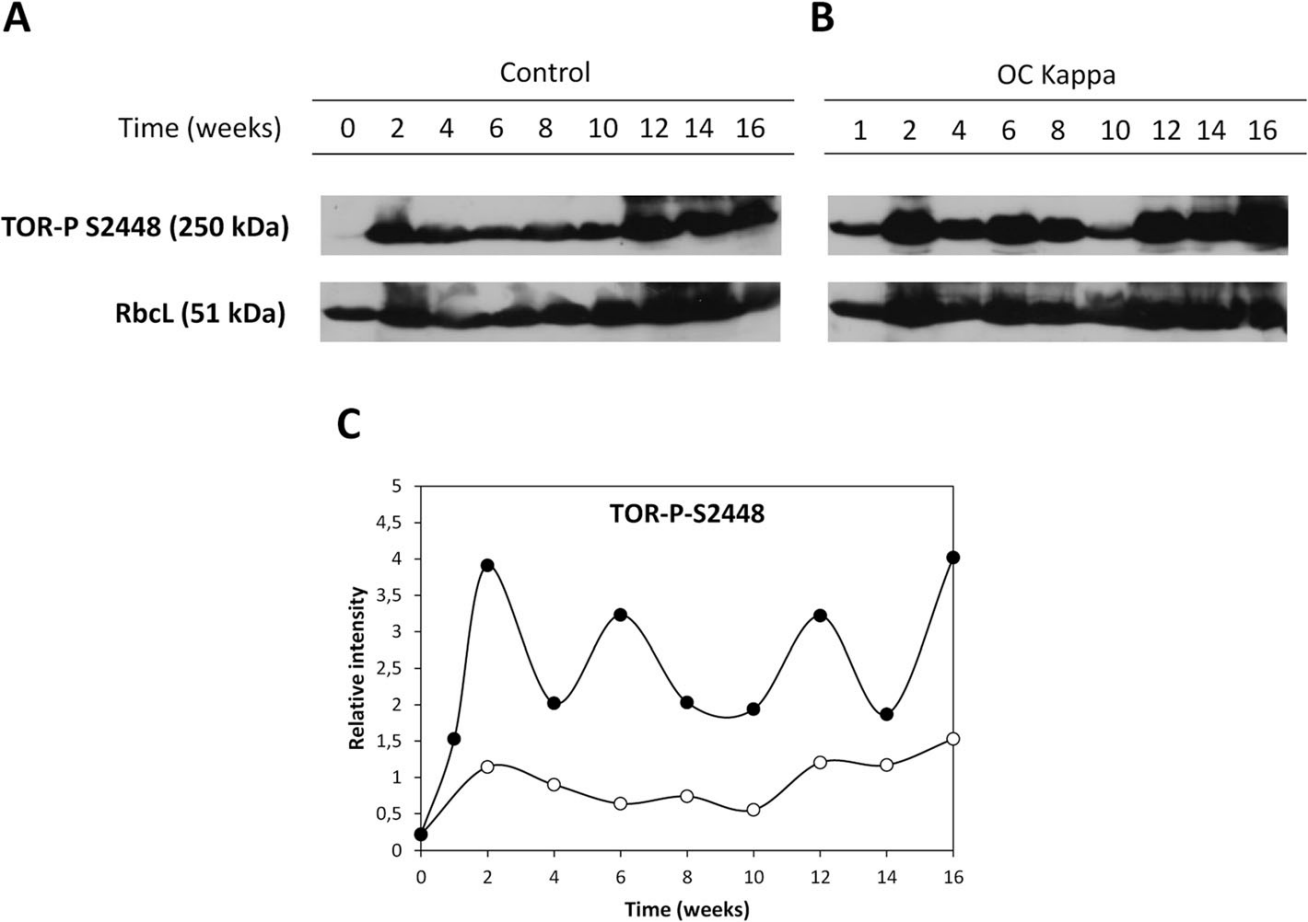
Detection of active TOR-P in *E. globulus* cultivated in control condition and trees treated with OC kappa. Level of active TOR kinase (TOR-P ser4448), and large subunit of ribulose-1,5-bisphosphate carboxylase/oxygenase (RbcL) in control (**a**) and in *E. globulus* trees treated with OC kappa at 1 mg mL^− 1^ (**b**). Levels of active TOR (**c**) in control (open circles) and treated trees (black circles) are expressed in relative units of band intensity corresponding to the ratio TOR/RbcL (**c**)

In order to analyze whether the increase in glucose and/or trehalose may induce the activation of TOR kinase, the level of TOR phosphorylated in ser2448 (TOR-P) as well as that of the large subunit (RbcL) of the enzyme ribulose-1,5-bisphosphate carboxylase/oxygenase (rubisco) were detected using specific antibodies (Fig. 3a). The level of active TOR was normalized using the level of RbcL (Fig. 3b). Trees treated with OC kappa showed a higher relative level of TOR-P from week 1 to the end of the experiment (week 21) and increases at weeks 1, 2, 6, 12 and 16 (Fig. 3a-c).

### OC kappa-induced increase in the levels of transcripts encoding proteins involved in photosystems and chlorophyll synthesis

**Fig. 4:**
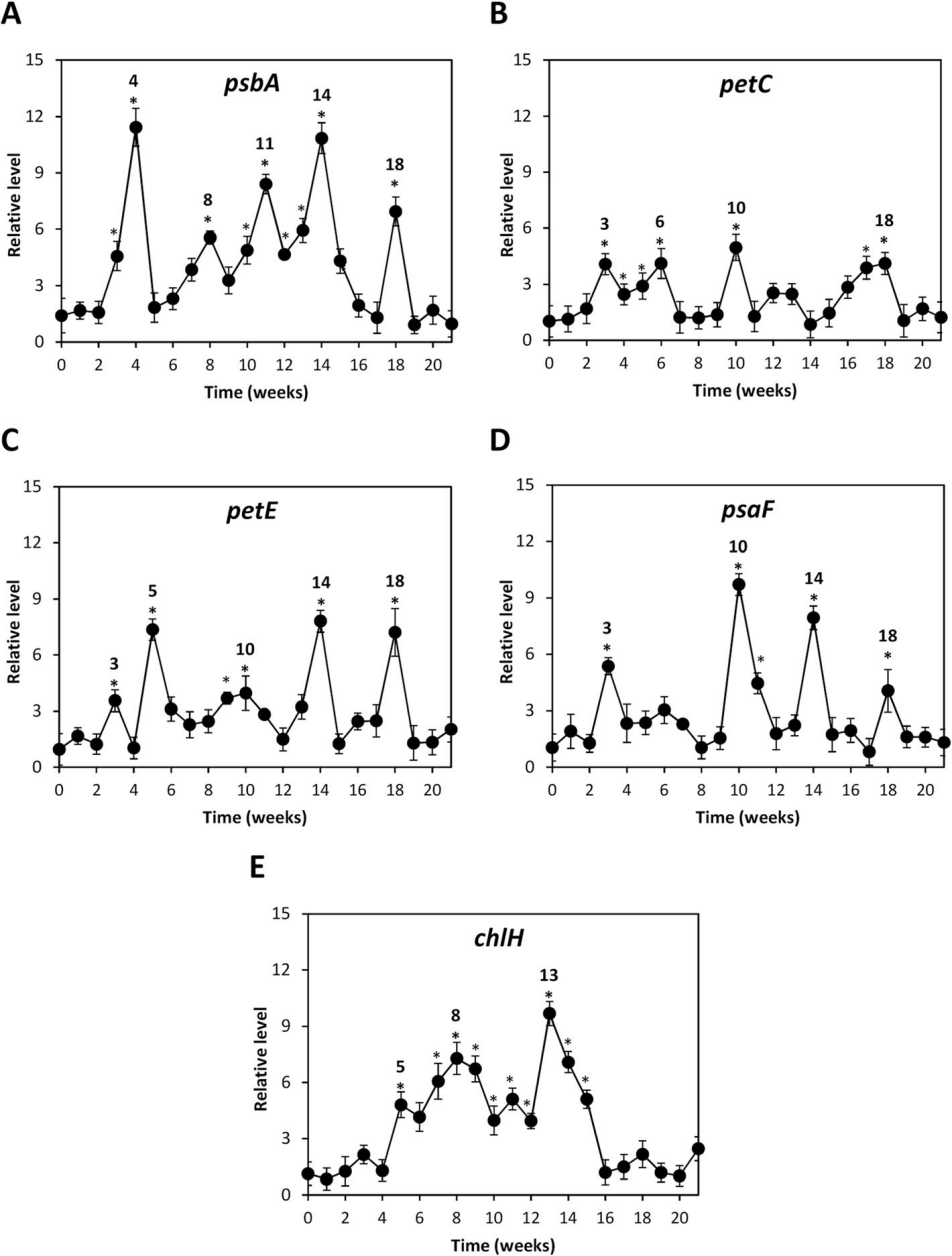
Level of transcript encoding proteins involved in photosynthesis in *E. globulus* treated with OC kappa. Relative level of transcripts encoding subunit A of photosystem II (*psbA*, **a**), subunit Rieske of cytochrome b6f *(petC*, **b**), plastocyanin (*petE*, **c**), subunit F photosystem I (*psaF*, **d**) and magnesium chelatase (*chlH*, **e**), in control (open circles) and *E. globulus* trees treated with OC kappa at 1 mg mL^− 1^ (black circles) Asterisks (*) represent significant differences (*p* < 0.05) between the level of transcripts at a certain week compared with the expression at the beginning of the experiments (week 0). The level of transcripts is expressed as 2^-ΔΔCt^. Circles represent mean values of three independent experiments ±SD

In order to analyze the reasons explaining the increase in net photosynthesis, the levels of transcripts encoding a subunit of photosystem (PS) II, *psbA*; the Rieske subunit of cytochrome b6f, *petC*; plastocyanin, *petE*; a subunit of PSI, *psaF*; and the enzyme magnesium chelatase involved in chlorophyll synthesis, *chlH*, were analyzed. Trees treated with OC kappa showed significant increase in the level of *psbA* transcripts at weeks 3–4, 8, 10–14 and 18 (Fig. 4a). Peaks of *petC* expression were observed to be significant at weeks 3–6, 10 and 17–18 (Fig. 4c). The increase of *petE* transcripts were significant at weeks 3, 5, 9–10, 14 and 18 (Fig. 4c). Expression of *psaF* was significantly higher at weeks 3, 10–11, 14 and 18 (Fig. 4d). Finally, *chlH* transcripts increased significantly from weeks 5 to 15 (Fig. 4e).

### OC kappa-induced increases in the level of transcripts encoding enzymes involved in basal metabolism

**Fig. 5:**
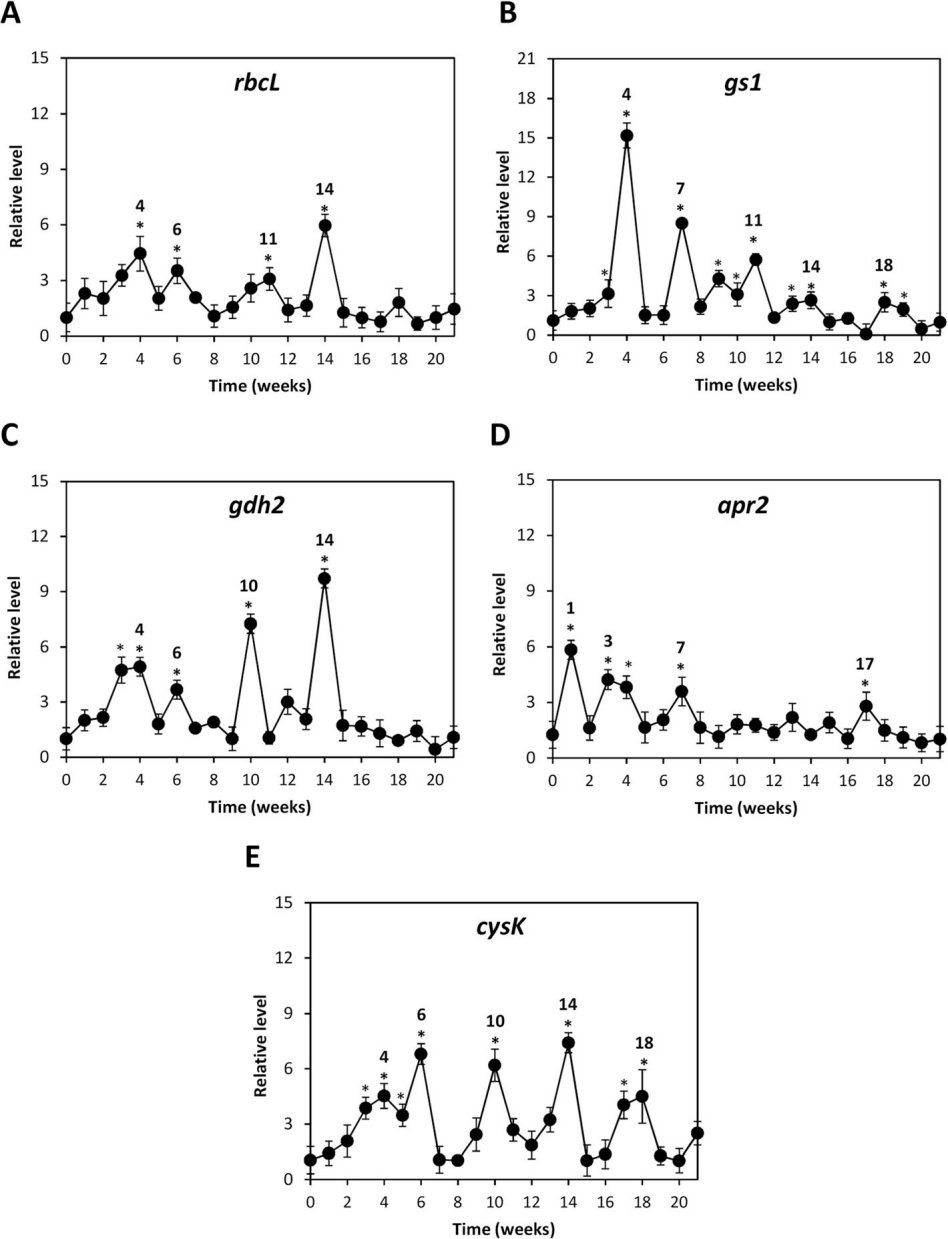
Levels of transcripts of enzymes encoding enzymes involved in basal metabolism in *E. globulus* treated with OC kappa. Relative level of transcripts encoding the large subunit of ribulose-1,5-carboxylase/oxygenase (r*bcL*, **a**), glutamine synthase (*gs1*, **b**), glutamate dehydrogenase (*gdh2*, **c**), 5′-adenilylsulfate reductase (*apr2*, **d**) and O-acetylserine thiol-lyase (*cysK*, **e**) in control (open circles) and *E. globulus* trees treated with OC kappa at 1 mg mL^− 1^ (black circles). Asterisks (*) represent significant differences (*p* < 0.05) between the level of transcripts at a certain week compared with the expression at the beginning of the experiments (week 0). The level of transcripts is expressed as 2^-ΔΔCt^. Circles represent mean values of three independent experiments ±SD

In order to analyze the reasons explaining the increase in activities of enzymes involved in C, N and S assimilation observed in previous works, the level of transcripts encoding the large subunit of the enzyme rubisco involved in C assimilation, *rbcL*; the enzyme glutamine synthase (GlnS) involved in N assimilation, *gs1*; the enzyme glutamate dehydrogenase (GDH) involved in N assimilation, *gdh2*; the enzyme 5′-adenilylsulfate reductase (APR) involved in S assimilation, *apr2*, and the enzyme O-acetylserine thiol-lyase (O-ASTL) involved in S assimilation, *cysK*, were detected. Treated trees showed a significant increase in *rbcL* transcripts at weeks 4, 6, 11 and 14 (Fig. 5a), in *gs1* at weeks 3–4, 7, 9–11, 13–14 and 18–19 (Fig. 5b), in *gdh2* at weeks 3–4, 6, 10 and 14 (Fig. 5c), in *apr2* at weeks 1, 3–4, 7 and 17 (Fig. 5d) and in *cysK* at weeks 3–6, 10, 14 and 17–18 (Fig. 5e).

### OC kappa-induced increase in the level of transcripts encoding enzymes involved in secondary metabolism

**Fig. 6:**
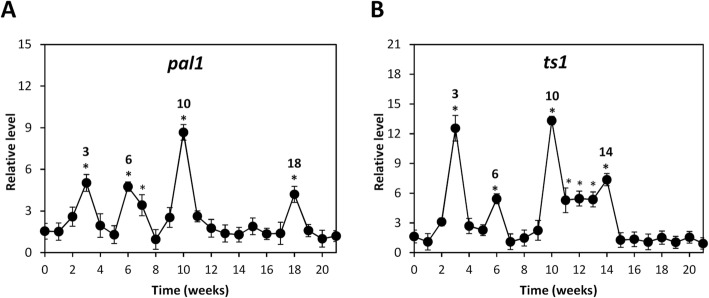
Level of transcripts encoding enzymes involved in secondary metabolism in *E. globulus* treated with OC kappa. Relative level of transcripts encoding phenylalanine ammonia-lyase (*pal1*, **a**) and terpene synthase (*ts1*, **b**), in control and in *E. globulus* trees treated with OC kappa at 1 mg mL^− 1^ (black circles). Asterisks (*) represent significant differences (*p* < 0.05) between the level of transcripts at a certain week compared with the expression at the beginning of the experiments (week 0). The level of transcripts is expressed as 2^-ΔΔCt^. Circles represent mean values of three independent experiments ±SD

In order to analyze the reasons explaining the increase in PPCs and terpenes induced by OC kappa and reported in previous works, the level of transcripts encoding enzyme phenylalanine ammonia-lyase (PAL) involved in PPCs synthesis, *pal*, and the enzyme terpene synthase involved in terpenes synthesis, *ts1*, were analyzed. Trees treated with OC kappa showed an increase in *pal1* transcripts at weeks 3, 6–7, 10 and 18 (Fig. 6a), and in *ts1* transcripts at weeks 3, 6 and 10–14 (Fig. 6b).

## Discussion

### OC kappa-induced increases in the level of glucose, trehalose and active TOR-P precede the increases in photosynthesis and growth

In this work, we showed that treatment of *Eucalyptus* trees with OC kappa induced an initial increase in the level of glucose at week 1, trehalose at weeks 1–3 and TOR-P at week 1–2. In this sense, it has been shown that the increase in glucose and fructose leads to an increase in G6P and G1P levels [31] as well as in T6P level in plants [51]. In addition, it was shown the increase in trehalose reflect the increase in T6P, an intermediate in trehalose synthesis, in rice plants [32]. Moreover, it was shown that G1P, G6P and T6P directly inhibit snRK1, a kinase that inhibit TOR [36] through phosphorylation of RAPTOR [11]. Thus, the increase in glucose and trehalose observed in *Eucalyptus* trees may lead to an increase in G1P, G6P and T6P inhibiting SnrK1 [36] and leading to the increase in TOR-P and the activation of TOR pathway.

In addition, OC kappa induced an increase in the level of transcripts encoding enzymes involved in glucose production such as F16BP and amylase at weeks 1–3 which explain the increase in glucose level observed at day 1. Additional increases in glucose were observed at weeks 9–11 and 17–19, in trehalose level at weeks 8–9, 12, 15–16 and 18–21 and in glucose-producing enzymes at weeks 7–8, 10–11 and 13–16. The latter suggests that the increase in glucose level observed at weeks 9–11 and 17–19 may be due, at least in part, to the increase in the level of transcripts of glucose-producing enzymes. On the other hand, an increase in net photosynthesis was observed at week 3 preceding the increase in growth observed at week 9 indicating that the initial increase in photosynthesis may contribute to stimulation of growth in *E. globulus* trees. Moreover, the increase in glucose and active TOR-P at weeks 1–2 precedes the increase in photosynthesis at week 3 suggesting that the increase in transcripts involved in photosynthesis is probably due to the activation of TOR pathway. In this sense it has been shown that the inhibition of TOR pathway using AZD8055 leads to a decrease in the level of transcripts encoding subunits of photosystems and enzymes of chlorophyll synthesis in *Arabidopsis* [39] indicating that TOR kinase is involved in the activation of gene expression. In this sense, it has been shown in yeast that TOR-P can enter into the nucleus and bind RNA polymerase II leading to activation of gene expression [40]. Thus. It is possible that TOR-P can also enter into the nucleus in plants allowing the increase in expression of genes involved in photosynthesis and basal metabolism leading to the stimulation of growth.

It is important to mention that *E.globulus* plants were treated with 50 *μ* M rapamycin or 250 *μ* M AZ8055 and they did not show growth inhibition (data not shown) but, at the contrary, they displayed an increased height compared to the controls. Consequently, *Eucalyptus* trees might have alternative pathway that is TOR-independent controlling growth. In this sense, it has been shown in *A. thaliana* that TOR activation inhibits autophagy. However, there is an alternative TOR-independent pathway that is activated by oxidative stress and endoplasmic reticulum stress that also inhibits autophagy [52]. Thus, *Eucalyptus* trees may present an alternative pathway that is TOR-independent that can also stimulate growth.

### OC kappa-induced increases in the level of transcripts encoding proteins of photosystems and chlorophyll synthesis showing an oscillatory pattern

It has been previously shown that *E. globulus* treated with OC kappa and cultivated until 4 months showed a persistent increase in net photosynthesis [47]. Here, we showed that OC kappa enhanced the levels of transcripts encoding proteins involved in photosynthesis. Thus, the persistent increase in net photosynthesis observed in *E. globulus* trees is due, at least in part, to an increase in gene expression. In addition, OC kappa induced and increase in total chlorophyll observed at weeks 3–11 and this increase partially overlaps with the higher expression of magnesium chelatase, a key enzyme in chlorophyll synthesis, which increases at weeks 5–15. Thus, the increase in expression of genes involved in chlorophyll synthesis may be partially responsible for the increase in total chlorophyll. It is important to mention that the increase in transcripts encoding PS proteins showed an oscillatory pattern with peaks at weeks 3–4, 5–8, 10, 14 and 18, as it was observed for transcripts encoding enzymes that produced glucose. In this sense, it has been shown in animal cells that TORC1 activity is controlled by the circadian clock and that a similar mechanism may occur in plants [3]. However, oscillations in the level of transcripts observed in this work are not circadian since they are observed along weeks. Thus, it is possible that a different mechanism is governing enhanced expression observed along weeks which may correspond to a “week clock”, different to the circadian clock, that is involved in the stimulation of photosynthesis and growth in *E. globulus* trees.

### OC kappa-induced increase in the level of transcripts encoding enzymes of basal and secondary metabolisms showing an oscillatory pattern

It has been previously observed that treatment with OC kappa increases activities of enzymes involved in C assimilation, rubisco; in N assimilation, GlnS and GDH; and in S assimilation, APR and O-ASTL in *E. globulus* [47]. In addition, results obtained in *E. gobulus* trees treated with OC kappa showed an increase in PPCs levels [46] as well as in volatile terpenes [50]. In this work, we determined that the level of transcripts encoding enzymes of basal metabolism as well as those involved in the synthesis of secondary metabolites are increased in response to OC kappa. Thus, the stimulation of basal and secondary metabolisms is due, at least in part, to the increase in gene expression. In this sense, it has been recently shown that an oligo-guluronic acid fraction obtained from alginates of marine brown algae bind to Toll-like receptor 4 (TLR4) in human macrophages activating TOR pathway and promoting proliferation and expression of pro-inflammatory cytokines which may lead to the stimulation of the immune response [53]. Thus, it possible that *E. globulus* cells may possess a receptor that bind OC kappa triggering the activation of TOR pathway leading to the activation of expression of enzymes of basal and secondary metabolisms which may result in the stimulation of growth and defense response in *E. globulus* trees.

In addition, we showed that OC kappa increased expression of genes involved in C, N and S assimilation presenting an oscillatory pattern with increases at weeks 3–4, 6–7, 10–11, 14 and 17–18. Moreover, the level of transcripts encoding enzymes involved in secondary metabolism, PAL and TPS showed increases at weeks 3, 5–6, 8–11, 13–14 and 17–19. As mentioned before, the oscillatory pattern in the level of transcripts encoding enzymes of basal and secondary metabolisms may be governed by a “week clock” different from circadian clock in plants.

## Conclusions

OC kappa induced an increase in net photosynthesis beginning at week 3 and a subsequent increase in height beginning at week 9 in *E. globulus* trees. In addition, OC kappa induced initial increases in the level of glucose at week 1, trehalose at week 1–3, and TOR-P at week 1–2. Furthermore, OC kappa induced an increase in the level of transcripts encoding proteins involved in photosynthesis, an enzyme related to chlorophyll synthesis, and enzymes involved in C, N and S assimilation, and associated with the syntheses of PPCs and terpenes, beginning at weeks 3–4. Interestingly, the increase in transcripts of the latter proteins showed an oscillatory pattern observed mainly at weeks 3, 6, 10–1, 16 and 18. Thus, OC kappa induce an increase in glucose and trehalose levels that may lead to an increase in G6P and T6P which may inhibit SnRK1 leading to the activation of TOR pathway. Thus, the stimulation of growth and defense against pathogens observed in *E. globulus* trees is due, at least in part, to the increase expression of genes involved in photosynthesis, and basal and secondary metabolisms and these increases may involve activation of TOR pathway.

## Methods

### Plant culture, treatment with OC kappa and measurement of height

*E. globulus* trees were obtained from seeds produced by Semillas Imperial S.A. (Los Angeles, Chile). Plants having an initial height of approximately 30 cm (*n* = 10 for each control and treated groups) were sprayed on leaves with water (control group) or with 5 mL of an aqueous solution containing OC kappa at a concentration of 1 mg mL^− 1^ once at the beginning of each week, four times in total, and cultivated outdoors in plastic bags containing composted soil for 17 additional weeks during spring and summer of 2015. Leaves (10 g) were obtained from the middle height part of control and treated trees, one day after each treatment, at the same time in the day (11 h in the morning), divided into three samples (*n* = 3) and frozen in liquid nitrogen for further analyses. The height of *E. globulus* trees were determined using measuring tape.

### Quantification of photosynthesis

Net photosynthesis were detected in five leaves located in the middle part of each control and treated *E. globulus* plants (*n* = 10) using a portable infrared gas analyzer Ciras-1 (PP systems, Hitchin, UK), a leaf cuvette of 12. 5 cm^2^ using a red/white LED light source, a photon irradiance of 1000 μmol quanta m^− 2^ s^− 1^ photosynthetic active radiation (PAR), a CO_2_ concentration of 500 ppm and a relative humidity of 70% at 24 °C for 1 min.

### Quantification of total chlorophyll

Quantification of chlorophylls *a* and *b* was performed as described in [54]. Fresh leaves (0.1 g) were frozen in liquid nitrogen and homogenized in a mortar with a pestle. One mL of acetone was added and the mixture was incubated at 4 °C for 90 min. The mixture was centrifuged at 14.000 rpm for 5 min using a micro-centrifuge. The supernatant was recovered and the absorbance determined at 665 and 649 nm using a Hewlett Packard/Agilent spectrophotometer model 8453 (Santa Clara, CA, USA). Total chlorophyll was calculated by addition of chlorophylls *a* and *b* and the concentration of chlorophylls was calculated using the following formula:

$$ \mathrm{Chlorophyll}\ a\left(\upmu \mathrm{g}\ {\mathrm{mL}}^{\hbox{-} 1}\right)=13.96\ {\mathrm{A}}_{665}-6.88\ {\mathrm{A}}_{649} $$

$$ \mathrm{Chlorophyll}\ b\left(\upmu \mathrm{g}\ {\mathrm{mL}}^{\hbox{-} 1}\right)=24.96\ {\mathrm{A}}_{665}-7.32\ {\mathrm{A}}_{649} $$

### Quantification of glucose

Fresh leaves (0.1 g) were frozen in liquid nitrogen and homogenized in a mortar. Five hundred μL of distilled water were added and the mixture centrifuged at 14.000 rpm for 5 min. The supernatant was recovered and an aliquot of 30 μL was added to 500 μL of glucose oxidase/peroxidase kit reaction mixture (Valtek Diagnostics, Santiago, Chile). The absorbance was determined at 505 nm and the concentration was calculated using a calibration curve prepared using glucose at concentrations of 0.2 to 2 mg mL^− 1^.

### Quantification of trehalose

Quantification of trehalose was performed as described in [55]. Fresh leaves (0.1 g) were frozen in liquid nitrogen and homogenized in a mortar. Two mL of ethanol were added; mixture was boiled for 1 h and ethanol was left to evaporate at 60° in an oven. Five mL of 5 mM sulfuric acid were added and the mixture was centrifuged at 3.200 rpm for 10 min. The supernatant was filtered through 0.2 μm pore PDVF filters and boiled in water for 1 h to hydrolyze sucrose. Once cold, the pH was neutralized with sodium hydroxide, the solution evaporated and the residue was dissolved in distilled water. A sample of 20 μL was analyzed by HPLC (Agilent 1100, CA, USA) with a Sugar Pack1 column (300 mm × 6.5 mm, Waters Corp., MA, USA) at 75 °C and coupled to a refraction index detector at 55 °C. The isocratic elution program consists of 40 min with a mobile phase 0.1% EDTA-calcium and a flux of 0.35 mL min-1 using a pressure of 38 bars. The calibration curve was prepared using trehalose at concentrations ranging 0 to 5 mg mL^− 1^.

### Preparation of protein extracts

Protein extracts were prepared as described in [56]. Fresh leaves (1 g) were frozen with liquid nitrogen and homogenized in a mortar. Three mL of extraction buffer (0.5 M Tris-HCl, 0.7 M sucrose, 1 mM PMSF, 50 mM EDTA, 0.1 M KCl and 0.2% β-mercaptoethanol pH 8.0) were added and the homogenate was shaken on ice for 10 min. One mL of phenol at pH 6.6–8.0 was added, the mixture was shaken on ice for 10 min and centrifuged at 3.200 rpm for 10 min at 4 °C. The organic phase was recovered and mixed with 4 volumes of 0.1 M ammonium acetate solubilized in methanol. The mixture was shaken using a vortex and incubated overnight at − 20 °C for protein precipitation. The mixture was centrifuged at 3.200 rpm for 15 min at 4 °C, and the protein pellet was washed twice with ammonium acetate at 0.1 M in methanol, and then once at the same concentration in acetone; the pellet was dried at room temperature and solubilized in 50 mM Tris-HCl pH 8.0. Proteins were quantified using Bradford reagent and the calibration curve was prepared using bovine serum albumin [57].

### Quantification of phosphorylated TOR (TOR-P)

Proteins (5 μg) were separated using a biphasic denaturant polyacrylamide gel (6% stacking phase and 12% resolving phase), and electrophoresis was performed at 110 V for 1.5 h. Proteins were electro-transferred to a nitrocellulose membrane using a TransBlot system (Bio-Rad) and 400 mA, at 4 °C for 1 h. The transfer of protein was verified by staining the membrane with Ponceau Red dye. The membrane was blocked with 5% skim milk solubilized in TTBS buffer (20 mM tris-HCl pH 7.5, 0.1 mM NaCl and 0.1% Tween 20), and washed three times with TTBS at room temperature for 10 min. The membrane was incubated with the monoclonal antibody anti-human TOR-P Ser2448 which binds to Ser2424 in *Arabidopsis* TOR (1:1000, Abcam ab109268) or anti-RbcL (1:2500, Agrisera AS03037) (Schepetelnikov et al., 2013) at room temperature for 1 h. The membrane was washed three times with TTBS at room temperature for 10 min, incubated with the secondary antibody anti-Rabbit IgG conjugated with HRP (Agrisera, AS09602) at room temperature for 1 h, and washed three times with TTBS at room temperature for 10 min. The membrane was incubated with a chemo-luminescent substrate (SuperSignal West Femto, Thermo Scientific, Rockford, IL, USA) for 5 min and was exposed to an X-ray film (Thermo Scientific, Rockford, IL, USA) for 3 min to detect TOR-P ser2448, or for 30 s to detect RbcL. Bands in the film were scanned and then quantified using Image Studio software (Li-Cor, USA).

### RNA extraction

Total RNA was extracted from *Eucalyptus* leaves as described in [58]. Fresh leaves (1 g) were frozen in liquid nitrogen and homogenized in a mortar with a pestle. Ten mL of solution A containing 100 mM Tris-HCl pH 8.0, 0.35 M sorbitol, 10% (w/v) polyethylenglycol 6000 and 2% (w/v) of β-mercaptoethanol were added and the mixture was shaken for 1 min. The mixture was centrifuged 3.500 rpm, at 4 °C for 15 min, and the supernatant was discarded. The pellet was solubilized in 10 mL of solution B containing 300 mM Tris-HCl pH 8.0, 25 mM EDTA, 2 M NaCl, 2% (w/v) CTAB, 0.05% (w/v) spermidine, 2% PVPP and 2% (w/v) β-mercaptoethanol; the mixture was heated at 65 °C and incubated at 65 °C for 10 min and shaken every 2 min using a vortex. A similar volume of a solution of chloroform/isoamyl alcohol (24:1) was added and the mixture was centrifuged at 3.200 rpm at 4 °C for 10 min. The aqueous phase was extracted once more with a similar volume of chloroform/isoamyl alcohol and centrifuged at 3.200 rpm at 4 °C for 10 min. The aqueous phase was recovered and total RNA precipitated by addition of 0.1 volume of 0.3 M sodium acetate, pH 5.2, and 0.6 volumes of isopropanol; the mixture was incubated at − 80 °C for 30 min. The mixture was centrifuged at 14.000 rpm, at 4 °C, for 20 min, and the supernatant discarded. The pellet was solubilized in 1 mL of nuclease free (DEPC-treated) water and total RNA was precipitated adding 0.3 volumes of 10 M lithium chloride; the mixture was incubated at 4 °C overnight. The mixture was centrifuged at 13.000 rpm, at 4 °C, for 30 min, and the supernatant was discarded. The pellet was solubilized in 0.1 mL of DEPC-treated water and total RNA precipitated adding 0.1 volume of 3 M sodium acetate pH 5.2, and 2 volumes of 70% cold ethanol; the mixture was centrifuged at 13.000 rpm at 4 °C for 20 min, and the supernatant discarded. The pellet was washed with 200 μL of 70% cold ethanol and centrifuged at 13.000 rpm at 4 °C for 10 min, and the supernatant discarded. The pellet was dried at room temperature and solubilized in 50 μL of DEPC-treated water. The concentration and purity of total RNA was determined measuring the absorbance at 260 and 280 nm, and in an agarose gel; RNA was stored at − 80 °C for further gene expression analyses.

### Quantification of transcript levels by qRT-PCR

The relative level of transcripts was quantified by qRT-PCR using a real-time thermocycler Rotorgene 6000 (Corbett, Australia). Transcripts involved in glucose accumulation and consumption: were those encoding fructose-1,6-bisphosphatase 1 (f*bp1*), a key enzyme in glucose synthesis; a-amylase 3 (amy3), an important enzyme in starch degradation and glucose production; ADP-glucose pyrophophorylase 1 (*apl1*), determinant enzyme in starch synthesis. Transcripts encoding photosystem proteins were: the subunit A of photosystem II (*psbA*), Rieske subunit of cytochrome b6f (*petC*), plastocyanin (*petE*), subunit A of photosystem I (*psaF*). Transcripts encoding enzymes were magnesium chelatase (*chlH*), a key enzyme of chlorophyll synthesis; the large subunit of ribulose-1,5-bisphosphate carboxylase/oxygenase (rubisco) (*rbcL*), a key enzyme in C assimilation; glutamine synthase (*gs1*), an enzyme involved in N assimilation; glutamate dehydrogenase (*gdh2*), an enzyme involved in N assimilation; O-acetylserine thiol-lyase (*cysK*), an enzyme involved in S assimilation; 5′-adenilylsulfate reductase (*apr2*), an enzyme involved in S assimilation; phenylalanine ammonia-lyase 1 (*pal1*), a key enzyme of phenylpropanoid pathway; and terpene synthase 1 (*ts1*), an enzyme involved in terpenes synthesis. RNA 18S was used as housekeeping transcript and its level did not change along weeks in control or treated plants (data not shown). PCR primers are listed in Additional file 1: Table S1. qRT-PCR reactions were performed using Sensimix One-step kit (Quantace, UK), 75 ng of total RNA, 200 nM primer solution and 3 mM magnesium chloride. Relative transcript level from three independent replicates was expressed as 2^-ΔΔCT^ [59]. To this end, mean values of control samples were subtracted to mean values of treated samples to determine fold-change in expression.

### Statistical analyses

Data were subject to one-way analysis of variance (ANOVA) and post hoc Tukey Test, previous to the evaluation of the requirements of normality and homogeneity of variance. Significant differences were estimated over 3 independent replicates at a 95% confidence interval.

## Additional file

Additional file 1: **Table S1.** Primers used to amplify cDNA from transcripts encoding enzymes involved in glucose synthesis, proteins involved in photosynthesis and enzymes of basal and secondary metabolisms by qRT-PCR. (DOCX 20 kb)

## Data Availability

All the experimental data is available at the online repository: https://figshare.com/s/1bd228e7ab5bdbc6427c

## Abbreviations

chlH: magnesium chelatase
cysK: O-acetylserine thiol-lyase
gdh2: glutamate dehydrogenase
gs1: glutamine synthase
*pal1*: phenylalanine ammonia-lyase 1
petC: Rieske subunit of cytochrome b6f
petE: plastocyanin
psaF: subunit A of photosystem I
psbA: subunit A of photosystem II
SnRK1: SNF-1-related kinase 1
TOR: Target of rapamycin
ts1: terpene synthase 1
apr2: 5′-adenilylsulfate reductase
rbcL: large subunit of ribulose-1,5-bisphosphate carboxylase/oxygenase (rubisco)
