# Supplementary material for: Oligo-carrageenan kappa increases glucose, trehalose and TOR-P and subsequently stimulates the expression of genes involved in photosynthesis, and basal and secondary metabolisms in Eucalyptus globulus
Source: BMC Plant Biol. 2019 Jun 17;19:258. doi: 10.1186/s12870-019-1858-z (PMC6580502; doi:10.1186/s12870-019-1858-z)
Supplement: Supplementary file 1 — Table S1. Primers used to amplify cDNA from transcripts encoding enzymes involved in glucose synthesis, proteins involved in photosynthesis and enzymes of basal and secondary metabolisms by qRT-PCR. (DOCX 20 kb) [file 12870_2019_1858_MOESM1_ESM.docx]

**Table S1**

| **Gene** | **Primer sequences** | | **Annealing**  **T (°C)** | **Product**  **(bp)** |
| --- | --- | --- | --- | --- |
| ***psaF*** (GI: 702489015)  Phosotystem I subunit F  (*Eucalyptus grandis*) | F  R | 5’–GCTCAAGAAGCTGGAGTCGT–3’  5’–TGCTTGCCGTAGTTGTCGAA–3’ | 60 | 114 |
| ***psbA*** (GI: 502172942)  Photosystem II subunit A  (*Cicer arietinum*) | F  R | 5’–AGAGAGACGCGAAAGCGAAA–3’  5’–GGGCAGCAATGAAAGCGATA–3’ | 57 | 191 |
| ***petC*** (GI: 702364431)  Cyrochrome b6f subunit Rieske  (*Eucalyptus grandis*) | F  R | 5’–GAAGGAAAGGTGCTGGTGGT–3’  5’–CCGTATGTTGCCAGCGTTTT–3’ | 60 | 71 |
| ***petE*** (GI: 702379677)  Plastocyanin  ( *Eucalyptus grandis*) | F  R | 5’–TGTTCAAGAACTACGCCGGG–3’  5’–GCCTTTCTCGGTCAAGGTCA–3’ | 60 | 152 |
| ***chlH*** (GI: 702247709)  Magnesium chelatase  (*Eucalyptus grandis*) | F  R | 5’–CTGCGCTCTCTGTTCGTTT–3’  5’–GCCTTGGGAGCTGGTAATGT–3’ | 59 | 132 |
| ***rbcL*** (GI: 844754)  Rubisco large subunit  (*Arabidopsis thaliana*) | F  R | 5’–GCATATGCCTGCTTTGACCG–3’  5’–TCGACTGCAAGATCACGTCC–3’ | 57 | 165 |
| ***gs1*** (GI: 104454001)  Glutamine synthase  (*Eucalyptus grandis*) | F  R | 5’–CCGGGATCAACATCAGTGGG–3’  5’–AAGTAAGGACCACCCCAGCA–3’ | 62 | 156 |
| ***gdh2*** (GI: 702251300)  Glutamate dehydrogenase  (*Eucalyptus grandis*) | F  R | 5’–CCGTGGCGTTGTTTTTGCTA–3’  5’–CAACTTTGCTGCCCAAGACC–3’ | 60 | 115 |
| ***cysK*** (GI: 357480874)  O-acetylserine thiol lyase  (*Medicago truncatula*) | F  R | 5’–TATGGAAAGGCACGGATGGG–3’  5’–AAGACACCAGGGACAAAGCC–3’ | 57 | 211 |
| ***apr2*** (GI: 702321790)  5’-adenilylsufate reductase  (*Eucalyptus grandis*) | F  R | 5’–CACAGATTGGCTTGTTCCGC–3’  5’–AACCGCTCGTAATCCTCCAC–3’ | 59 | 199 |
| ***pal1*** (GI: 30687012)  Phenylalanine ammonia lyase  (*Arabidopsis thaliana*) | F  R | 5’–ACACAAGAGCAACGGAGGAG-3’  5’–TCCTTCTGAAGTGCGACACC-3’ | 58 | 221 |
| ***ts1*** (GI: 702490249)  Terpene synthase  (*Eucalyptus grandis*) | F  R | 5’–AAGCGGTGACAAAAGCGTTC–3’  5’–CCACAGCATTGAGGTCCCAT–3’ | 59 | 123 |
| ***fbp1*** (GI: 565388105)  Fructose 1,6-bisphosphatase  (*Solanum tuberosum*) | F  R | 5’–GCAAGTATCCCGCTGATGGT–3’  5’–TTTCCCGTTGGGGCTTTTCT–3’ | 59 | 134 |
| ***amy3*** (GI: 702421945)  α-amylase  (*Eucalyptus grandis*) | F  R | 5’–TGCCGCATTGGAAAGATGTG–3’  5’–CCAATGACCCTGGGTAGAGC–3 | 55 | 136 |
| ***apl1*** (GI: 30687199)  ADP-glucose pyrophosphorylase  (*Arabidopsis thaliana*) | F  R | 5’–GTTTCCCCACAGCAAACGAC–3’  5’–TGATGGTGGCAGGTTTCTCC–3’ | 59 | 218 |
| ***rRNA18S*** (GI: HO048251.1)  Ribosomal RNA 18S  (*Eucalyptus grandis*) | F  R | 5’–GTGCACAAAATCCCGACTCT–3’  5’–GCGATCCGTCGAGTTATCAT–3’ | 58 | 168 |
